# Mitochondrion-Permeable Antioxidants to Treat ROS-Burst-Mediated Acute Diseases

**DOI:** 10.1155/2016/6859523

**Published:** 2015-11-16

**Authors:** Zhong-Wei Zhang, Xiao-Chao Xu, Ting Liu, Shu Yuan

**Affiliations:** ^1^College of Resources Science and Technology, Sichuan Agricultural University, Chengdu 611130, China; ^2^College of Bioindustry, Chengdu University, Chengdu 610106, China; ^3^Sichuan Kelun Pharmaceutical Co. Ltd., Chengdu 610071, China

## Abstract

Reactive oxygen species (ROS) play a crucial role in the inflammatory response and cytokine outbreak, such as during virus infections, diabetes, cancer, cardiovascular diseases, and neurodegenerative diseases. Therefore, antioxidant is an important medicine to ROS-related diseases. For example, ascorbic acid (vitamin C, VC) was suggested as the candidate antioxidant to treat multiple diseases. However, long-term use of high-dose VC causes many side effects. In this review, we compare and analyze all kinds of mitochondrion-permeable antioxidants, including edaravone, idebenone, *α*-Lipoic acid, carotenoids, vitamin E, and coenzyme Q10, and mitochondria-targeted antioxidants MitoQ and SkQ and propose astaxanthin (a special carotenoid) to be the best antioxidant for ROS-burst-mediated acute diseases, like avian influenza infection and ischemia-reperfusion. Nevertheless, astaxanthins are so unstable that most of them are inactivated after oral administration. Therefore, astaxanthin injection is suggested hypothetically. The drawbacks of the antioxidants are also reviewed, which limit the use of antioxidants as coadjuvants in the treatment of ROS-associated disorders.

## 1. Introduction

Endogenous reactive oxygen species (ROS) were produced in cells over time, causing oxidative-damage to nucleic acids, protein, lipids, and other cellular components. ROS are now considered as signalling molecules to change the expression of a large number of genes [1]. The relationship between some diseases and oxidative-damage has been well studied. A large number of reports showed that oxidative stress is correlated with the pathogenesis of multiple age-related diseases, like cancer and neurodegenerative diseases, and several other common diseases such as ischemia-reperfusion injury, stroke, hypertension, heart failure, atherosclerosis, diabetes, rheumatic diseases, and Alzheimer disease [2–10]. Therefore, a lot of antioxidants have been adopted to prevent and alleviate disease-accompanying oxidative-damage. However, some human clinical data of antioxidant therapeutics indicated negative or ambiguous results or insignificant benefits. Even some antioxidants showed apparent side effects [10, 11]. In this review, we compare representative mitochondrion-permeable antioxidants through analyzing their therapeutic mechanisms, the application ranges, and side effects.

## 2. ROS-Burst-Mediated Acute Diseases

### 2.1. ROS Burst in Ischemia-Reperfusion

When blood supply or oxygen supply returns to the ischemic tissue, the reperfusion injury occurs. In this condition, restoration of blood flow and oxygen supply does not restore cellular normal functions but induces inflammation and oxidative-damage [3, 12].

Reperfusion of ischemic tissues is usually accompanied with microvascular damage, which increases capillary and arteriole permeability and leads to fluid filtration and diffusion. These damaged endothelial cells generate more ROS but less nitric oxide after reperfusion, and the disequilibrium induces subsequently inflammatory responses [3, 12, 13]. At the same time, leukocytes, circulated with the newly returning blood, release interleukins, free radicals, and other inflammatory factors, which damage the tissue further [3, 12, 13]. The reintroduced oxygen damages nucleic acids, enzymes, and the plasma membrane. Oxidative-damaged cellular membrane may release more ROS in turn. Then ROS may also trigger redox signalling indirectly and the subsequent cell death or apoptosis. Leukocytes may also bind to the small capillary endothelium, causing more ischemia [3, 12, 13].

### 2.2. ROS Burst in Avian Influenza Infections

The infection of avian influenza virus (AIV) results in multiple complications to the patient, causing multiorgan failures and may be associated with the excessive immune responses, which may be the main reason for its high pathogenicity and mortality [2, 10]. The AIV infection induces a cytokine storm, including chemokines, interferon-inducible protein IP-10, interferon *β*, and interleukin-6 (IL-6) and cell death presumably [14–17]. Investigations suggested that healthy young people with stronger immune system may become a main target of AIV attacks [2].

In our previous review of the drugs to avian influenza infection, a large drug combination (including antioxidants, protectant of mitochondrial membrane permeability, immunomodulators, protease inhibitors, and antiviral drugs) is proposed, which mainly focuses on cytokine control and may greatly reduce the mortality rate hypothetically [2]. For the drug combination, antioxidant is the most important medicine suggested, because of the fact that ROS play a crucial role in the inflammatory response and cytokine outbreak [18]. Neutrophil aggregation and oxidative-damage to alveolar epithelial membrane result in acute respiratory distress syndrome (ARDS) finally. The activated neutrophils induce a ROS burst (more than ten times explosion).

There are many similarities between pulmonary ischemia-reperfusion and AIV infection-induced ARDS. Both of the oxidative injuries include the following: (i) lipid peroxidation and oxidative-damage to cytomembrane and the organelle-membrane; (ii) enzyme activity inhibition; (iii) lysosomal protease releasing; and (iv) chemoattractant generation and aggregation of more neutrophils [19]. Thereby free radicals form a self-amplification feedback loop [18].

H1N1 infection also inhibits patient's catalase expression, therefore causing the hydrogen peroxide accumulation [20], while H5N1 triggers extracellular calcium influx, which induces apoptosis [21].

Major inflammatory response is the mitochondrial dysfunction. AIV infection or ischemia-reperfusion induces calcium overload and mitochondrial permeability transition (mPT). Apoptosis indicator cytochrome c is released from mitochondria. Cyclosporine A (CsA) can prevent this mitochondrial permeability transition and the subsequent apoptosis [22]. CsA-treated cells are protected from ischemia-reperfusion injury, but not from tumour necrosis factor *α* (TNF*α*) or Bax (Bcl-2 associated X protein) induced cell death [22]. Both ROS-mediated apoptotic pathway and NF-*κ*B-mediated survival pathway are activated by the TNF*α*. ROS accumulation facilitates the cell-death pathway [23]. The ratio of proapoptotic protein Bax to antiapoptotic protein Bcl-2 is also regulated by the ROS level. Superoxide anion induces the survival pathways, while hydrogen peroxide triggers the cell-death pathways [24]. Thus antioxidants may block both TNF*α* and Bax-mediated apoptosis pathways [2].

### 2.3. Vitamin C May Be Potentially Used as the Candidate Antioxidant to Treat Avian Influenza Infections

In view of the advantages such as relatively effective, nontoxic, and easy to be absorbed, ascorbic acid (vitamin C, VC) was suggested as the candidate antioxidant for avian influenza infections [2]. VC scavenges free radicals through a nonenzymatic process. In the 19th century, VC was used to cure cold (influenza infection), encephalitis, hepatitis, and some other viral diseases for over a hundred years [25–27].

An investigation indicated that 50% of H5N1-infected patients in Vietnam did not die. Ely [28] found that the survivals may take large amounts of VC from their foods, which may alleviate the inflammatory responses.

Influenza patients need 4.4 g or higher levels of VC to control the virus or alleviate the symptom [25–28]. However, the common oral dosage of VC tablets is 100–300 mg a day, much lower than the influenza treatment requires. Oral intakes of VC that exceed 1 g may cause side effects, like vomiting, stomach cramps, diarrhea, and nausea [25–28]. Therefore the VC injection should be used for AIV infections. Nevertheless, high doses are still required. Additionally, long-term use of high level of VC (>2-3 g a day) may result in scurvy after VC administration is stopped [25–27]. These drawbacks should be considered before the clinical therapies.

### 2.4. Other ROS-Related Airway Disorders, Chronic Obstructive Pulmonary Disease, for Example

Chronic obstructive pulmonary disease (COPD) is a major and rapidly increasing health problem associated with a chronic inflammatory response, predominantly in small airways and lung parenchyma. Oxidative stress induced by reactive oxygen species and nitrogen species plays a central role in the pathophysiology of COPD [29]. At the subcellular level, mitochondrial dysfunction (accompanied with a decreased mitochondrial membrane potential) in patients with COPD is associated with excessive mitochondrial ROS levels, which contribute to enhanced inflammation and cell hyperproliferation. Thus, targeting mitochondrial ROS represents a promising therapeutic approach in patients with COPD, such as the mitochondria-targeted antioxidant MitoQ (see later discussion of MitoQ) [30].

## 3. Mitochondrion-Permeable Antioxidants

The most pivotal aspects of antioxidant therapies are the site concentration effects. Antioxidant efficiency is fully dependent on the locus concentration, since, as many other pharmaceutical compounds, antioxidants also have their “pharmacological windows.” Therefore, these scavenging/quenching compounds should concentrate in the target-tissue (or subcellular site) in order to efficiently remove exceeding ROS without eliminating essential redox signalling molecules, such as nitric oxide, hydrogen peroxide, S-Nitrosoglutathione (GSNO), and nitro/nitrosyl-lipid peroxides [24, 31]. It is well known that cellular redox status defines the fate of one cell. Depending on the redox status, eukaryotic cells could proliferate, keep it in steady state (G0 phase), or enter into cell death, either by apoptosis (moderate oxidative condition; intrinsic mitochondrial pathway) or by necrosis (high oxidative insults) [24, 31]. More interestingly, the redox status sensibility varies obviously upon the cell type that hepatic cells are more plastic than neurons [32]. Therefore, the biggest challenge researchers have nowadays on prescribing antioxidant therapies is how to reach the proper antioxidant concentration* in situ* for a precise redox modulation against a ROS-mediated pathology.

**Figure 1:**
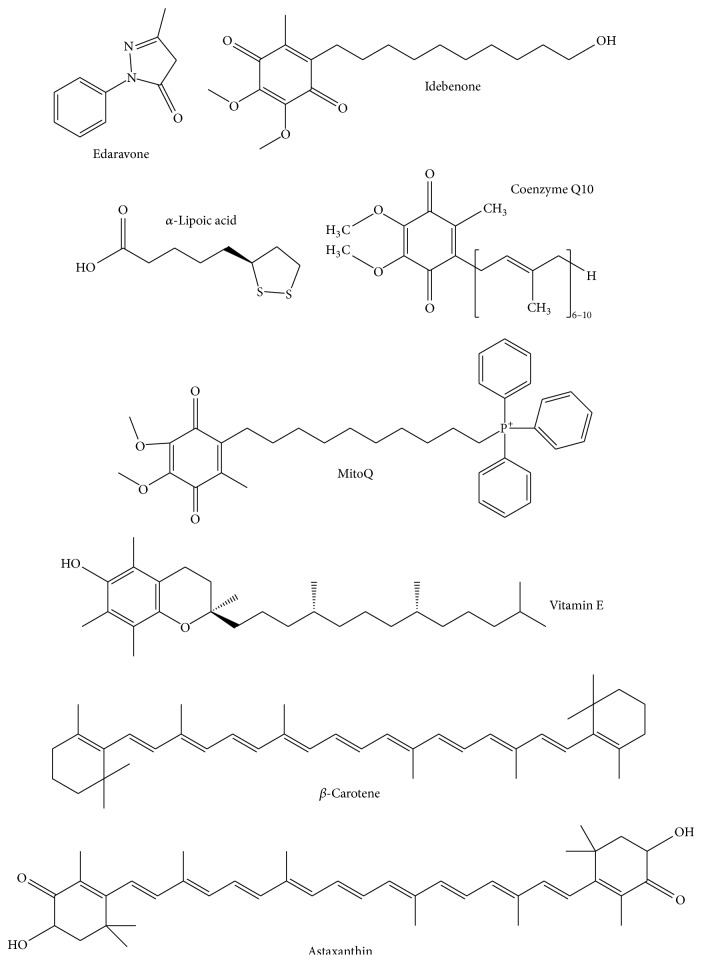
Chemical structures of representative mitochondrion-permeable antioxidants (edaravone, idebenone, *α*-Lipoic acid, coenzyme Q10, MitoQ, vitamin E, *β*-carotene, and astaxanthin).

As discussed above, ROS-burst-mediated mitochondrial dysfunction and mitochondrial-derived apoptosis play a crucial role in the inflammatory response during avian influenza infection or ischemia-reperfusion. Thus for these ROS-burst-mediated acute diseases, mitochondrion-permeable antioxidants should be much more effective than water-soluble antioxidants (like VC). Edaravone, idebenone, *α*-Lipoic acid, carotenoids (especially astaxanthin), vitamin E, coenzyme Q10, and mitochondria-targeted antioxidants MitoQ and SkQ are summarized as follows (Table 1 and Figure 1). Interestingly, most of them contain a six-membered carbon-ring with a long alkyl side chain and multiple hydroxyl groups and aldehyde groups (Figure 1). All of them are liposoluble. Therefore, they could traverse across the cell membrane and the mitochondrial membrane and accumulate in mitochondria. On the contrary, most water-soluble antioxidants are distributed in the cytosol (Figure 1).

### 3.1. Representative Mitochondrion-Permeable Antioxidants

Edaravone (3-methyl-l-phenyl-pyrazoline-5-one) has been approved in Japan since 2001. Edaravone can reduce ischemic-stroke-induced neuronal damage [33]. However, there are also studies that do not approve the effects. Even some cases of nephrotoxicity were reported for edaravone [34].

Idebenone (2,3-dimethoxy-5-methyl-6-(10-hydroxydecyl)-1,4-benzoquinonenoben) is a short chain benzoquinone, structurally similar to coenzyme Q10. Idebenone functions as an antioxidant and electron carrier [35]. Although idebenone has some effects on Alzheimer's diseases [35, 36], the solid clinical evidences are still missing. Therefore, its clinical application is limited [37]. The most common side effects are gastrointestinal complaints and some level of neurotoxicity or cardiotoxicity [38].

*α*-Lipoic acid (LA) is a unique lipid and water-soluble antioxidant. It is a naturally occurring dithiol compound and essential for mitochondrial bioenergetic process [39]. LA and its reduced-form dihydrolipoic acid are important mitochondrion-permeable antioxidants. LA has been approved for diabetic neuropathy treatment [39, 40].

Carotenoids, consisting of over 600 lipid-soluble plant pigments and a few water-soluble carotenoids (such as crocin), are present in many fruits and vegetables. The common carotenoids include *α*-carotene, *β*-carotene, lycopene, *β*-cryptoxanthin, lutein, and zeaxanthin [41]. Among them, *β*-carotene, the vitamin A precursor, has been most well studied. They neutralize free radicals effectively [42]. However, there are inconsistent conclusions about the role of *β*-carotene in cardiovascular diseases (CVD) prevention [43]. Moreover, a study indicated that high intake of carotenoids resulted in a faster skeletal muscle breakdown (skeletal muscle integrity reducing) [44]. Astaxanthin is a peculiar carotenoid, which will be discussed in detail later.

Among the vitamin E family, *α*-tocopherol is the most predominant form. The hepatic *α*-tocopherol transfer protein binds and carries *α*-tocopherol to all body's cells [45]. Most *α*-tocopherol is associated with lipoproteins, scavenging LCOO^•^ results and inhibiting low-density lipoprotein (LDL) oxidation. Thus, *α*-tocopherol is thought to have a role in atherosclerosis prevention. The uptake process of oxidized LDL by the macrophage scavenger receptor and the foam cell formation are blocked by the *α*-tocopherol treatment [46]. However, some reports did not support the protective role of vitamin E in prostate cancer [47, 48].

Coenzyme Q10 (CoQ10), with its oxidized-form ubiquinone and reduced-form ubiquinol, is an endogenous lipid, which participates in the mitochondrial electron transport in the respiratory chain [49]. CoQ10 has been used to treat a variety of diseases, such as cardiovascular diseases [50], migraine [51], hypertension [52], and neurodegenerative diseases [53]. Although CoQ10 is considered a safe drug, further large-scale studies are still needed to show its clinical usefulness.

MitoQ was designed in the late 1990s as a mitochondria-targeted antioxidant by Kelso et al. [54]. Both MitoQ and coenzyme Q10 belong to the ubiquinone components. The ubiquinone structure of MitoQ can be activated in the mitochondrion (by the mitochondrial respiratory complex II) to form the ubiquinol antioxidant. Thus, MitoQ increases the mitochondrial antioxidant capacity* in situ* and thereby decreases mitochondrial oxidative-damage [54].

MitoQ is a lipophilic molecule bearing a cation moiety, which makes it pass directly through the mitochondrial membrane, because of the fact that the component is positively charged (a hydrophobic structure) [55]. Therefore, MitoQ is an effective mitochondria-targeted antioxidant.

The ability of MitoQ and the mitochondrial oxidative-damage after the treatments (oral or intraperitoneal administration) have been studied in the mouse model. The following diseases have been studied: Alzheimer's disease, hypertension, type I diabetes, heart attack, sepsis, fatty liver disease, alcohol-induced steatohepatitis, doxorubicin, and cocaine cardiotoxicity [56, 57]. These findings are consistent with the conclusion that mitochondrial oxidative-damage is the potential therapeutic target in multiple diseases and pathologies.

However, for Parkinson's disease trials, MitoQ did not show a benefit, maybe because of the irreversible neuronal damage in patient's brain cells [58]. Therefore, more studies of MitoQ in humans are much needed. Moreover, only successful phase II assessments of oral MitoQ tablets were reported. It is not a FDA-approved drug so far.

SkQ (10-(6′-plastoquinonyl)decyltriphenyl-phosphonium) also is organic molecules composed of a large number of organic cations attached with a plastoquinone. SkQ traverses across the cellular membranes and accumulates in mitochondria. The level of a penetrating cation in mitochondria can be more than 1000-fold higher than its extracellular level [59]. Therefore, it is another mitochondria-targeted antioxidant.

Several studies indicated that SkQ protects cells from age-related diseases efficiently, including cataract, retinopathy, glaucoma, balding, canities, osteoporosis, hypothermia, and torpor [59]. However, its safety and the clinical usefulness need further investigations. Like MitoQ, SkQ is also not a FDA-approved drug so far.

### 3.2. Astaxanthin Is a Promising Antioxidant

Better than above antioxidants, here we introduce another one, astaxanthin, to be a candidate drug for AIV infection cure and some other diseases (Table 1). Astaxanthin, a dietary carotenoid, is present in algae, shrimp, lobster, crab, salmon, and some other organisms [60–64]. Its antioxidant activity is far exceeding the existing antioxidants. The ROS-scavenging capacity is 6000 times that of VC, 800 times that of coenzyme Q10, 550 times that of VE, 200 times that of polyphenols, 150 times that of anthocyanins, and 75 times that of *α*-Lipoic acid [65]. Most importantly, no apparent side effects or negative results have been reported for astaxanthin [60–62]. In leukocyte cells, half of the total astaxanthin is distributed in the mitochondria. Astaxanthin is also distributed in microsomes and nuclei [66]. Therefore, it is a mitochondrion-permeable antioxidant.

Natural astaxanthin plays an important role in preventing atherosclerosis. Low-density lipoprotein (LDL) oxidation is the main reason of atherosclerosis. Astaxanthin treatment increased high-density lipoprotein (HDL) significantly and reduced LDL effectively, while *β*-carotene or canthaxanthin has no such effect. The main reason may be that only astaxanthin can reduce apolipoprotein oxidation, therefore being important for preventing arteriosclerosis, cardiovascular diseases, and ischemic brain damage [67, 68].

Astaxanthin also maintains the eyes and central nervous system healthy. Retina contains high levels of unsaturated fatty acids and oxygen supply. The singlet oxygen is generated in the retina upon high-energy light illumination. However, for mammals, carotenoids in diet are enough to maintain eye health and can quench these free radicals [69]. Recent study indicated that astaxanthin can pass through the blood-brain barrier and prevent retina cell oxidation [70]. Astaxanthin also has a good effect on preventing and treating macular degeneration [70].

Astaxanthin is an anti-inflammatory and pain reliever, blocking different biochemical factors that cause ouch and pain [71]. More specifically, astaxanthin inhibits cyclooxygenase 2 (COX2) enzyme activities, which are related with many diseases, such as osteoarthritis, rheumatoid arthritis, dysmenorrhea, and acute pain [72]. Astaxanthin and Celebrex (another COX2 inhibitor) work cooperatively for some diseases, which therefore were suggested to be taken both together to alleviate oxidative-damage [72].

Astaxanthin affects not only the COX2 signalling pathway but also multiple cytokines, like nitric oxide, interleukin 1-*β*, prostaglandin E2, C-Reactive Protein (CRP), NF-*κ*B, and TNF*α* [72]. A study also showed that astaxanthin is a useful antioxidant to treat insulin resistance by protecting cells from TNF*α* and palmitate-induced oxidative-damage [73]. Recent study suggested that astaxanthin also inhibits apoptosis in alveolar epithelial cells via mitochondrial ROS signalling pathways, also indicating its mitochondrial location [74].

Astaxanthin also activates T-cell and inhibits autoimmune reactions [75]. The risk of many different types of cancer can be significantly reduced by dietary intake of astaxanthin along with other carotenoids [76–78]. In the mouse breast cancer model, astaxanthin treatment caused higher levels of apoptotic cancer cells and protective interferons, inhibiting tumor growth [79]. (i) Astaxanthin prevents cancer initiation by alleviating DNA oxidative-damage [80, 81]. (ii) Astaxanthin promotes early check and elimination of cells undergoing malignant transformation by activating immune surveillance [82]. (iii) Astaxanthin prevents cancer cell growth in cells by boosting immune detection [83, 84]. (iv) Astaxanthin inhibits rapid tumor cell growth by blocking tumor cell reproductive cycle and inducing tumor cell apoptosis [85–87]. (v) Astaxanthin prevents tumor cell spreading by decreasing tumor cell's tissue-melting proteins [84].

McNulty et al. [88] studied membrane structures of carotenoids and the relationship to their biological activities. They found that the vertical orientation of astaxanthin in membranes may be crucial for its high efficiency on removing aggressive free radicals from membranes, especially in the presence of water-soluble antioxidants, such as glutathione and/or ascorbic acid [88].

## 4. Side Effects of Antioxidants

Most of the so-called antioxidant compounds also develop prooxidant properties under specific conditions, such as ascorbic acid (>1 mM) that induces Fe(III) reduction to Fe(II) [89]. Epigallocatechin 3-gallate (EGCG) produces hydrogen peroxide and hydroxyl radicals in the presence of Fe(III) [90].

Clinical trials of some antioxidants in humans showed negative or ambiguous results or insignificant benefits [10, 11, 91–95]. The reasons may be as follows. (i) Oxidative-damage is neither the primary cause nor the only cause of the disease. (ii) Patients do not benefit from the same antioxidant treatment equally. (iii) Some antioxidants by oral administration are of lower efficiencies. (iv) Some antioxidant molecules have toxic effects that mask their ROS-scavenging activities. (v) Certain antioxidants are not effective in well-nourished populations [11, 95].

On the other hand, ROS accumulation does not always correlate with disease onsets positively. Watson [96] postulates that diabetes (especially the type 2 diabetes), dementias, cardiovascular disease, and some cancers may develop, when oxidative redox potential in the endoplasmic reticulum is too low to form normal disulphide bonds [97–99]. Maintaining a certain level of ROS may be necessary for correct protein folding with disulphide bonds, which may be associated with type 2 diabetes and Alzheimer's disease or some other diseases [96, 100, 101]. Thus, the antioxidants may produce negative or ineffective impacts on some diseases.

Interactions of carotenoids (such as canthaxanthin) with the lipid membranes and the aggregation of this pigment may be the factors enhancing canthaxanthin toxicity towards the macula vascular system, which leads to the further development of canthaxanthin retinopathy [102]. And high and long-term beta-carotene supplementation may increase lung tumor rates in heavy smokers [103].

## 5. Hypothesis of Astaxanthin Injection

Side effects of antioxidants (antioxidant-induced stress) only present when antioxidants overwhelm the body's free radicals [11]. Thus, antioxidants should be used carefully for chronic diseases, such as diabetes and Alzheimer's disease, when cellular ROS levels are not particularly high (no ROS bursts occur). However, for ROS-burst-mediated acute diseases, such as avian influenza infection and ischemia-reperfusion, antioxidants should be used as early as possible to avoid or retard excessive immune responses. Mitochondrion-permeable or mitochondria-targeted antioxidants are preferred.

Astaxanthin is a good candidate drug for these acute diseases. However, so far, astaxanthin is not a clinical drug but merely a health care product. Most studies showed that its treatment effects are not as good as people expect, contrasting to its extraordinary high antioxidant activities [61]. One of the reasons may be that astaxanthin is usually applied by oral administration (such as astaxanthin soft capsule). All nourishments and oral drugs are digested in the gastrointestinal tract and then absorbed into gastric veins and intestinal veins and transported to the liver through the portal vein. Then, after the liver's process, they are transported throughout the body via heart and arteries. Astaxanthin is easy to be oxidized that most of them are inactivated during the digestion, absorption, and transportation. After avian influenza virus infection, for instance, severe oxidative-damage occurs at the lungs, where neither oral VC tablets nor oral astaxanthin capsules could reach effectively. For the same reason, active (reduced) astaxanthin could not reach atherosclerosis sites, retina, or brain arteries ideally too. Therefore the injection approach of astaxanthin may be adopted to these patients. It is well known that vitamin E injection (a mixture of oil for injection and VE) is better absorbed by the body since it goes directly into the blood stream [60, 62]. And recent studies indicated that VC injections have strong anticancer effects, especially when intravenous glutathione or vitamin K3 was applied synergistically [104, 105]. A similar astaxanthin injection could be easily developed. For AIV infections, astaxanthin by injection can be quickly absorbed and go directly into the pulmonary alveoli, where inflammatory reactions occur, through the body's blood circulation system.

It is well known that water- and lipid-soluble antioxidants act in synergism to efficiently remove aggressive radicals from hydrophobic compartments and, thereby, inhibit lipid peroxidation, which is extremely harmful to most organelles [106]. The collaborative mechanism involving *α*-tocopherol through tocopheryl formation and ascorbic acid has been studied since the middle of the 90s [106]. In other words, by combining lipid-soluble antioxidants (such as astaxanthin) with water-soluble ones (such as ascorbic acid) in lower concentrations, higher efficiency on ROS removal may be expected. However, cellular ROS should not be removed entirely for retaining the essential redox signalling molecules. The precise dosages need further investigations.

The astaxanthin injection might be suitable for other kinds of disease-accompanying oxidative-damage and inflammations. However, the astaxanthin injection must be subjected to clinical trials and FDA approval. Nevertheless, they are time-consuming processes. Before FDA approval, oral astaxanthin capsules are still suggested for AIV-infected patients. Because when most patients are identified as having avian influenza infections, they have been sick for several days, near or after the time pulmonary symptom developed. Their alveolar cells may become damaged. Thus the risk to develop acute respiratory distress syndrome (ARDS) is very high. So, for the general public, timely oral administration of antioxidants before the diagnosis in the hospital is very important [2, 107]. No matter if infected with avian influenza or common influenza, the patient is recommended to take VC (800–1000 mg a day presumably) or/and astaxanthin (24–48 mg a day presumably) before the hospital examination.

## 6. Conclusions

Considering the adverse effects of antioxidants, antioxidant drugs should be used carefully for chronic diseases, especially for diabetes and Alzheimer's disease, when a certain level of ROS is required for normally cellular functions. However, for ROS-burst-mediated acute diseases, mitochondrion-permeable antioxidants should be used in the early stage.

To treat ROS-accompanying diseases, no matter chronic or acute, antioxidants should be used combined with other therapeutic drugs. However, drug combinations may have additive or possible antagonistic effects on the disease development. And the dosage of the single compound should be adjusted according to the combination. Thus, carefully pharmaceutic studies should be done before certain antioxidant (e.g., astaxanthin injection) can really enter the clinical trial to oxidative-damage-related illnesses.

## Figures and Tables

**Table 1:** Licensed antioxidants for alleviating disease-related oxidative-damage. Their evidenced clinical uses, drawbacks, and possible side effects are summarized.

Drug's name	Clinical uses	Drawbacks	Possible side effects
Edaravone	Ischemic stroke	Limited testing and sometimes ineffective	Nephrotoxicity [95]

Idebenone	Alzheimer disease	Limited testing and sometimes ineffective	Gastrointestinal complaints, neurotoxicity, and cardiotoxicity [95]

*α*-Lipoic acid	Diabetic neuropathy and eye-related disorders	Limited testing and sometimes ineffective	Headache, tingling, skin rash, or muscle cramps [95]

Carotenoids	Inflammation, cancer, and cardiovascular diseases	Sometimes ineffective	Damage to skeletal muscle integrity (high-dose) [44], canthaxanthin retinopathy [102], and lung cancer in heavy smokers [103]

Vitamin E	Inflammation, cancer, and cardiovascular diseases	Sometimes ineffective	Hemorrhage and vitamin K deficiency (high-dose) [45]

Coenzyme Q10	Heart failure, migraine, hypertension, and neurodegenerative diseases	Limited testing, insoluble in water, therefore in low bioavailability, and sometimes ineffective	Largely gastrointestinal complaints (very high-dose) [50]

MitoQ	Alzheimer's disease, Parkinson's disease, hypertension, diabetes, heart attack, sepsis, alcohol-induced steatohepatitis, and cocaine cardiotoxicity	Sometimes ineffective in human bodies	No side effect observed (even after a long-term oral administration) [56]

SkQ	Age-related diseases	Limited testing	No side effect observed [59]

Astaxanthin	Atherosclerosis, coronary heart disease and ischemic brain damage, age-related macular degeneration, acute pain, inflammation, cancer, and cardiovascular diseases	Insoluble in water and sometimes ineffective	No side effect observed [60–64]
